# Biomaterials constructed for MSC-derived extracellular vesicle loading and delivery—a promising method for tissue regeneration

**DOI:** 10.3389/fcell.2022.898394

**Published:** 2022-08-25

**Authors:** Yu Lu, Yu Yang, Shiyu Liu, Shaohua Ge

**Affiliations:** ^1^ Shandong Key Laboratory of Oral Tissue Regeneration, Shandong Engineering Laboratory for Dental Materials and Oral Tissue Regeneration, Department of Biomaterials, School and Hospital of Stomatology, Cheeloo College of Medicine, Shandong University, Jinan, China; ^2^ State Key Laboratory of Military Stomatology, National Clinical Research Center for Oral Diseases, Shaanxi International Joint Research Center for Oral Diseases, Center for Tissue Engineering, School of Stomatology, The Fourth Military Medical University, Xi’an, China

**Keywords:** mesenchymal stem cells, extracellular vesicles (EVs), engineered vesicles, delivery systems, tissue regeneration

## Abstract

Mesenchymal stem cells (MSCs) have become the preferred seed cells for tissue regeneration. Nevertheless, due to their immunogenicity and tumorigenicity, MSC transplantation remains questionable. Extracellular vesicles (EVs) derived from MSCs are becoming a promising substitute for MSCs. As a route of the MSC paracrine, EVs have a nano-sized and bilayer lipid-enclosed structure, which can guarantee the integrity of their cargoes, but EVs cannot obtain full function *in vivo* because of the rapid biodegradation and clearance by phagocytosis. To improve the efficacy and targeting of EVs, methods have been proposed and put into practice, especially engineered vesicles and EV-controlled release systems. In particular, EVs can be cell or tissue targeting because they have cell-specific ligands on their surfaces, but their targeting ability may be eliminated by the biodegradation of the phagocytic system during circulation. Novel application strategies have been proposed beyond direct injecting. EV carriers such as biodegradable hydrogels and other loading systems have been applied in tissue regeneration, and EV engineering is also a brand-new method for higher efficacy. In this review, we distinctively summarize EV engineering and loading system construction methods, emphasizing targeting modification methods and controlled release systems for EVs, which few literature reviews have involved.

## Introduction

Extracellular vesicles (EVs) were first discovered in the 1960s and were previously described as cellular excrements (Wolf, 1967; Anderson, 1969); in recent decades, they have been recognized as intracellular communication mediators. EV refers to all lipid bilayer-enclosed extracellular particles, which were derived from cells and cannot self-replicate, as Minimal Information for Studies of Extracellular Vesicles (MISEV) defined in the latest guideline (Thery et al., 2018). EVs’ nanoscale size and capacities for transporting cellular components have attracted the attention of researchers (van Niel et al., 2018; Mathieu et al., 2019) and their important roles in physiological and pathological processes are gradually being revealed, especially in immunoregulatory and cancer processes (Raposo et al., 1996; Gyorgy et al., 2011). In addition to that, researchers have long focused on the regenerative effect EVs possess.

Mesenchymal stem cells (MSCs) have become preferable seed cells for tissue regeneration and because of their multi-lineage differentiation potential and secretory function, they can facilitate multiple tissue repair through their proliferation, homing, and paracrine function (Brown et al., 2019; Fu et al., 2019). However, a study showed that MSCs implanted in subjects withered in 48 h (Liu et al., 2018), which showed that the by-products rather than MSCs act as determinants in tissue regeneration. Moreover, MSC implantation can lead to local inflammation (Shi et al., 2010) and undirect cell differentiation (Pittenger et al., 1999; Shi et al., 2010), and recipients suffer from undesired teratoma (Zhao et al., 2011; Wang et al., 2014).

In recent years, EVs derived from MSCs (MSC-EVs) have attracted extensive attention. Owning to their nano-size, EVs were verified to realize key molecules’ targeted delivery *via* the lipid bilayer membrane and transmembrane ligand (Hu et al., 2021), which facilitates targeted tissue repairing. However, the mechanisms of tissue repair *in vivo* mediated by MSC-EVs are complicated and under study, including local immuno-environment modulation (She et al., 2020; Xiong et al., 2021), angiogenesis enhancement (Li et al., 2021a), inhibition of apoptosis (Yuan et al., 2022), and reduction of fibrosis (Cruz and Rocco, 2020). The high capacity of tissue repair makes MSC-EVs a promising part of tissue regeneration therapies, especially in biomaterial construction. The reasons include 1) EVs, as an endogenetic biological agent, have innate host affinity and can deliver easy-to-deactivate and -degrade substances to target cells. 2) The nano-size of EVs is suitable for traveling through the circulatory system and barriers (Dabrowska et al., 2019), which provides the possibility for distant delivery to promote specific organ regeneration. Nevertheless, as experiments processed, the hollow nanospheres showed a high clean-up ratio by the liver and kidney (Wiklander et al., 2015); they cannot maintain effective concentration in the tissue nor the circulation system, and local injection was mainly limited by their unsatisfying retention ratio. To solve this problem, researchers have established multiple biomaterial carriers for EV control-releasing, which are capable of continuous and effective functioning, such as bio-macromolecular hydrogel, electrostatic spinning scaffold, and membrane and polymerized sponge. They could retain vesicles in local tissues for a longer time with varying degrees of design and manufacture (Gu et al., 2021). Beyond that, researchers are attempting to improve EV targeting by constructing engineered vesicles. By altering transcripts of the donor cells, the membrane proteins enriched on the surface of vesicles can be artificially manipulated, for example, CD47 on EV surfaces can be increased to attenuate the degradation and inactivation of vesicles by the mononuclear phagocytic system (Chiangjong et al., 2021; Du et al., 2021). Researchers mostly improve the efficiency of EV treatment by the aforementioned methods, either by continuously releasing EVs or reducing the loss in circulation.

The purpose of this study is to review the functions of EVs generated by MSCs, and we intensively review recent literatures focused on tissue regeneration by incorporating MSC-EVs with biomaterials to summarize the suitable carriers for MSC-EVs, and explore novel methods for MSC-EV-based biomaterial construction.

## MSC-EVs participate in different regeneration processes

MSCs refer to stromal cells derived from post-natal populations which own abilities including self-renewal and multilineage differentiation potential. Nowadays MSCs as potential seed cells have been isolated from bone marrow (Pittenger et al., 1999), adipose tissue (Brennan et al., 2017), umbilical cord blood (Erices et al., 2000), placenta (Botelho et al., 2017), and dental tissues (Miao et al., 2006). MSCs share some common surface biomarkers (CD44, CD73, CD90, CD105, etc.), and have tri-lineage differentiation potential (Gan et al., 2020). But MSCs derived from different origins have their unique secretion profiles, which determine their application scene in tissue repairing and regeneration.

### EV classification

**FIGURE 1:**
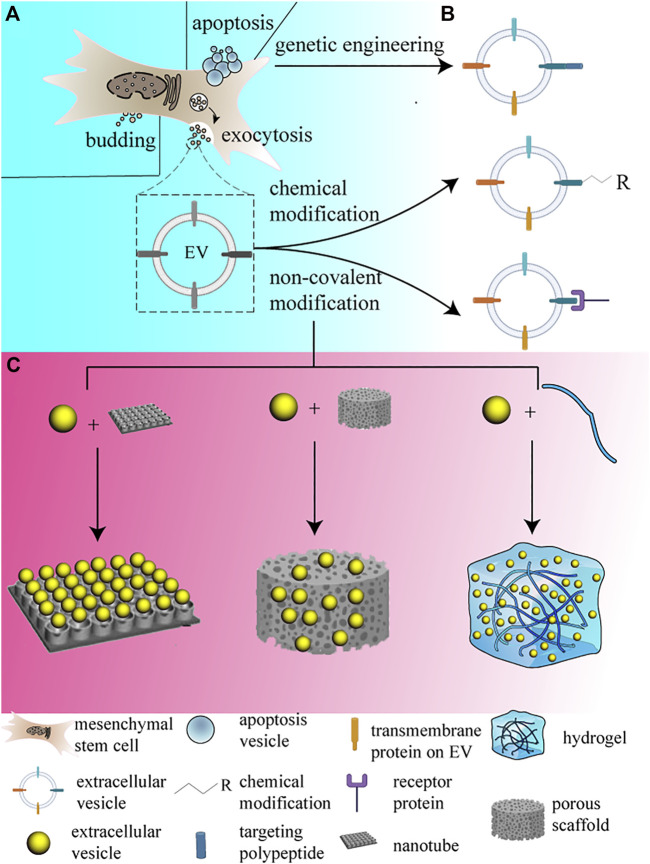
EVs and their engineering and loading strategies. The figure displays the routine approach EV secreted from MSCs, and lists the mentioned modification methods simply, and the loading methods are demonstrated simultaneously. **(A)** EVs are routinely secreted by budding and exocytosis, which corresponds to different sizes of EVs, EV secreted by budding commonly possesses larger size, while EVs secreted by exocytosis is usually smaller. Otherwise, apoptosis vesicles only emerge during apoptosis happens. **(B)** EVs can be modified in different methods, including genetic engineering, chemical modification and non-covalent modification. **(C)** EVs can be loaded onto different forms of biomaterials, they are shown from left to right relatively.

The EV is an important pathway through which MSCs perform secretory functions. According to their diameters, EVs can be classified as small-size EVs (sEVs), medium-size EVs (mEVs), and large-size EVs. The biogenesis of the sEVs occurs initially with the formation of early endosomes from endocytoses of the cell membrane; the early endosomes then become endosomes or multivesicular bodies and they begin to accumulate intraluminal vesicles, which are either degraded by lysosomes or released as exosomes into the extracellular space (van Niel et al., 2018). The larger vesicles’ biogenesis occurs *via* the direct budding of the cell membrane and releasing into the extracellular space; moreover, apoptosis vesicles are also large-size vesicles whose diameter ranges from 1–5 μm, but they originate only from apoptosis cells (Wang et al., 2021; Zheng et al., 2021) (Figure 1A). As research is advancing, EVs have been shown to contain functional peptides, nucleotides, small RNAs, lipids, and some metabolites, which can affect cellular functions and phenotypes at small doses. EVs mainly deliver the cargoes to their target cells *via* four conventional approaches: 1) EV phagocytosis; 2) membrane fusion; 3) endocytosis (lipid-raft, caveolin, and clathrin-mediated); and 4) macropinocytosis (Mulcahy et al., 2014). The way EVs enter the cells determines the mechanisms by which EVs affect cellular functions and the ultimate destiny of the cell. Correspondingly, EVs from different MSC derivations show diverse regenerative capacities, which can be contributed to the cellular or tissue specificity of EVs, and the different cargoes they carry determine their route in circulation and the final result.

### EVs derived from different mesenchymal stem cells have potential in tissue regeneration

Due to their self-renewal and multi-linage differentiation ability, stem cells are the preferred seed cells in regenerative medicine. They function as a precursor and are not only a substitute for dead cells but bear a regulating capacity in homeostasis maintenance and microenvironment improvement. MSCs are commonly isolated from adult tissues, and most of them are mesodermal-origin precursor cells embedded in adult tissues during developmental processes. The EV from these carries multiple key molecules such as nucleic acid and proteins; the enclosed nucleic acid and protein intercellular horizontal transfer can directly change the phenotype and function of the recipients. MSC-EV-mediated cell-free therapy has been demonstrated and verified in animal experiments, which could alleviate apoptosis, necrosis, boost the proliferation of renal tubular epithelial cells (Ding et al., 2011; Bruno et al., 2017), and reduce hepatic fibrosis (Hyun et al., 2015; Keshtkar et al., 2018). The different effects of EVs from different MSC sources on tissue regeneration are collected and summarized in Table 1. Although MSC-EVs of one derivation do not necessarily own explicit effects on one peculiar, the choice preference is probably related to the MSC-EV’s derivation. As a key mediator of MSC paracrine, the EV carries crucial information on tissue regeneration.

**TABLE 1:** Different regenerative effects of MSC-EVs from distinct derivation.

MSC	EV	Bone regeneration	Nerve regeneration	Vessel regeneration	Cardiac regeneration	Skin wound healing	Immunoregulation
BMMSC	Exosome/mEV/apoptosis vesicle	√(1, 2)	√(3)	√(2, 4)	√(5)	√(6, 7)	√(8-10)
ADMSC	Exosome	√(11)	√(6)	√(7, 12)		√(13)	√(14)
UCMSC	Exosome	(15)	√(16)	√(17)	√(18)	√(19, 20)	√(21)
PMSC	Exosome		√(22)	√(23, 24)		√(25)	
DPSC	Exosome	√(26)		√(27-29)			√(30, 31)
SHED	Exosome	√(32)	√(33, 34)	√(34, 35)			
PDLSC	sEV	√(36)					√(37)
SCAP	Exosome			√(38)		√(38)	
DFSC	sEV	√(39)					√(40)
GMSC	sEV		√(41)			√(42)	
iPSC-MSC	Exosome	√(43, 44)	√(45)	√(46)	√(47)	√(48, 49)	√(50-52)
ESC-MSC	Exosome	(53)					

BMMSC, bone marrow mesenchymal stromal cell; ADMSC, adipose mesenchymal stem cell; UCMSC, umbilical cord mesenchymal stem cell; PMSC, placenta mesenchymal stem cell; DPSC, dental pulp stem cell; SHED, stem cell from exfoliated deciduous teeth; PDLSC, periodontal ligament stem cell; SCAP, stem cell from apical papilla; DFSC, dental follicle stem cell; GMSC, gingival mesenchymal stem cell; iPSC, induced pluripotent stem cell; ESC, embryonic mesenchymal stem cell; sEV, small extracellular vesicle.

However, MSCs extracted from adult tissues have significant heterogeneity, which can lead to distinct effects among different donors and cell passage times (Chen et al., 2022). ESCs are single cells biopsied from eight-cell stage embryos, which are highly specialized pluripotent cells (Hur et al., 2021). Beyond that, induced pluripotent stem cells (iPSCs) reprogrammed using nonintegrating vectors also possess brilliant renewal and differentiation capacity. Both ESCs and iPSCs can be induced into mesenchymal linage, which evades the teratomas while inherenting the proliferative capacity (Brown et al., 2014). As for MSC-EVs, studies have shown that EVs from iPSC-MSCs and ESC-MSCs possess obvious pro-regenerative and immunoregulative effects. Xia et al. (2020) demonstrated that EVs from iPSC-MSCs could enhance angiogenesis by inhibiting vascular epithelial autophagy in ischemic stroke. Qi et al. (2016) also validated the promotion of bone regeneration *via* enhanced angiogenesis. EVs derived from iPSC-MSCs can even suppress the activation of immune cells and the expression of proinflammation factors (Hai et al., 2018; Feng et al., 2021), which are also mediated by EVs from ESC-MSCs (Wang et al., 2017a).

Apart from the EVs’ cellular derivations, cell culture conditions also determine the therapeutic effect of MSC-EVs. Accumulating pieces of evidence suggest that EVs secreted by MSCs pre-treated with chemical agents, hypoxia, and inflammatory microenvironment can significantly improve their pro-regenerative property. It is not only about MSC differentiation, survival, and homing capacities but also paracrine capacity. Ge et al. illuminated that MSCs pre-treated with hypoxia could enhance angiogenesis; Wei et al. showed that MSCs pre-treated with glycyrrhetinic acid significantly reinforced the therapeutic effect of their EVs in liver regeneration (Wang et al., 2017a). Moreover, Fu et al. demonstrated that MSCs pre-treated with oridonin-produced EVs were elucidated to activate autophagy instead of apoptosis in myocardial ischaemia/reperfusion cardiac cells (Wei et al., 2020). In addition, Ti et al. found that MSC-derived EVs preconditioned with lipopolysaccharide (LPS) had a more significant regulatory effect on macrophage polarization, which turns out to enhance diabetic cutaneous wound healing (Fu et al., 2021). Song et al. found a similar phenomenon that MSCs pre-treated with pro-inflammatory factor IL-1β could enhance the therapeutic effect of MSC-EVs in ameliorating symptoms of murine sepsis (Ti et al., 2015).

### Mechanisms of MSC-EVs promote tissue regeneration

MSC-EVs could promote tissue regeneration by various mechanisms, and they carry and transfer distinct bio-cargoes, which mirror their parental cells’ genomic and proteomic pools. But owing to the nano-scale of EVs, the cargoes they carry which significantly affect recipients are supposed to be the key genetic materials. As Akbari et al. reviewed, MSC-EVs act as a depot for the encapsulation of bioactive molecules to deliver them to the desired cells to function rather than enzymatic degradation (Song et al., 2017). MSC-exosomes contain more than 150 miRNAs (Akbari et al., 2020) and 850 proteins (Chen et al., 2010), and the key molecule delivery through EVs to target cells can lead to favorable phenotype changes. For example, exosomes derived from MSCs bear several cytokines and growth factors, including interleukin (IL) -6, IL-10, and transforming growth factor (TGF) β1, which regulate the local immune microenvironment (Lai et al., 2012). In addition, EVs derived from umbilical cord mesenchymal stem cells (UMSCs) are verified to promote angiogenesis and prevent scar formation in skin wound recovery; TGF-β/SMAD signaling is validated to be blocked by miR-21, miR-23a, miR-125b, and miR-145 enriched in EVs (Burrello et al., 2016). Also, the additional complement of MSC-EVs enhanced oligodendrogenesis, neurogenesis, and neural remodeling in the ischemic boundary region; EVs containing miR-133b (Burrello et al., 2016) and miR-17–92 clusters (Brown et al., 2014) can be transferred to astrocytes and neuron cells and consequently contribute to neurite remodeling and promote recovery.

MSC-EVs could shuttle their distinct bio-cargoes between cells; the cargos they carry somehow mirror their parental cells’ genomic and proteomic pools. In addition, the other non-coding RNAs can also induce transcriptomic changes of recipient cells *via* the EVs’ horizontal transfer. Huang et al. introduced lncRNA H19-enriched MSC-EVs which significantly improved cardiac function and promoted blood vessel formation; Huang et al. (2020) demonstrated that exogenous lncRNA H19 promotes the expression of miR-675-3p, miR-675-5p, VEGF (vascular endothelial growth factor), and ICAM (intercellular adhesion molecule) in vascular endothelial cells, which alleviate the ECs’ apoptosis and cultivate cardio-remodeling (Fang et al., 2016). Mao et al. illuminated that exosome-transported circRNA_0001236 could enhance chondrogenesis and suppress cartilage degradation *via* the miR-3677-3p/Sox9 axis.

MSC-EVs may function in the following ways:

(1) MSC-EVs can modulate the autophagic flux of recipient cells to facilitate tissue regeneration.

As a nano-scale cellular by-product, MSC-EVs can be easily taken up by phagocytes and non-phagocytic cells. The foreign vesicles were likely to change the phenotypes of recipients, which may be induced by an autophagic flux change. Debnath et al. found that EVs were strongly lysosome-associated (Mao et al., 2021) and they could correspondingly influence the autophagic flux of recipients (Leidal et al., 2020). Either activation or inhibition of autophagy can be related to tissue regeneration. Kuang et al. proved that adipose mesenchymal stem cell (ADMSC)-EVs rescued neurons under oxygen and sugar deprivation and promoted regeneration by inhibiting autophagic flow. Their work focused on microRNA transmission (miR-25-3p) by AMSC-EVs through the p53-Bcell lymphoma 2–interacting protein 3 (BNIP3) signaling axis, which significantly promoted the recovery of neurological functions in mouse apoplexy models (Salunkhe et al., 2020). In addition, Rong et al. elaborated that EVs derived from neural stem cells could reduce neuronal apoptosis, inhibit neuroinflammation, and promote functional recovery in spinal cord injury by increasing autophagic flux (Kuang et al., 2020).

(2) MSC-EVs could reverse pro-inflammatory macrophages to anti-inflammatory phenotypes by transmitting anti-inflammatory substances to immune cells. Nakao et al. verified that EVs derived from gingival mesenchymal stem cells (GMSCs) enhanced M2 macrophage polarization and inhibited periodontal bone loss. They also found that the application of CD39/73-enriched MSC-EVs could enhance the polarization of macrophages toward the M2 phenotype, which directly alleviated the local inflammatory environment (Rong et al., 2019). The mechanism is that the CD39-CD73-adenosine axis is necessary for immunoregulation, which could inhibit the proliferation of CD4^+^ T cells and promote tissue remodeling activity (Nakao et al., 2021). CD39 is associated primarily with endothelial cells and immune cell populations and it is known to be an ecto-nucleoside triphosphate diphosphohydrolase which could convert extracellular ATP into AMP (Nakao et al., 2021). CD73 (ecto-5′-nucleotidase, Ecto5′NTase) commonly expressed on the cytomembrane of MSCs (Antonioli et al., 2013) could dephosphorylate AMP into adenosine (Nakao et al., 2021). AMP accumulation could induce local inflammation, and the AMP/adenosine ratio could significantly affect the inflammatory microenvironment (Sanchez-Abarca et al., 2016).
(3) MSC-EVs may affect the energy metabolism of host cells. The phenomenon that the engulfed vesicles were mostly enriched in the mitochondrial area suggests that MSC-EVs can target the recipient cells’ mitochondrial metabolism pathways. Proteomic and RNA-seq analyses demonstrated that it can be achieved by the modulatory effect of several contained miRNAs, proteins, enzymes, and kinases involved in glycolysis such as glyceraldehyde-phosphate dehydrogenase (GAPDH), glucose-6-phosphate isomerase, in the tricarboxylic acid cycle (2-oxoglutarate dehydrogenase), and electronic transport chain (ATPase) (Loussouarn et al., 2021; Schneider et al., 2021). MSC-EVs have been reported to stimulate ATP production and the antioxidant defense of tubular epithelia cells through the activation of the KEAP1-NRF2 signaling pathway (Hogan et al., 2019), and they could transfer miR-222 in mesangial cells and induce miR-21 downregulation, which correspondingly rescue the function of electron transport chain complex, and relieve mitochondrial disorders (Cao et al., 2020).

However, the energy metabolic signal may not only be transferred by exosomal nuclei but also by exosomal organelles. In recent studies, Clair et al. proved that EVs released from stress-induced cells are enriched with oxidatively damaged mitochondria, which can induce a burst of ROS in cardiac tissue which protects the heart through hormesis (Gallo et al., 2016). Meanwhile, Gentaro et al. verified that EVs enriched with mitochondria significantly improved post-MI cardiac function through the restoration of bioenergetics and mitochondrial biogenesis. To be specific, the EV-enclosed mitochondria could fuse with the recipients’ endogenous mitochondrial network, retrieve ATP production, and improve contractile profiles of hypoxia-injured iCMs (Crewe et al., 2021).

## EV engineering for enhancing targeting

To improve the therapeutic efficiency of MSC-EVs, researchers came up with strategies that modified EV surfaces by chemical or genetic engineering to achieve a targeting effect on specific cells. The targeting modifications improve the efficiency of EV uptake by specific cell lineages, reduce the injected doses, and enhance the therapeutic effects. The EVs’ surface modification is aimed at conferring cell-type targeting specificity on EVs, and there are three common ways for EV surface modification **(** Figure 1B).

### Genetic engineering

The expression of targeted peptides on EV surfaces is achieved by inserting motifs of targeting proteins or polypeptides right behind EV surface membrane proteins in a protein-encoding sequence (Figure 1B). This strategy can ensure that the targeting peptides or proteins are expressed on EV membranes, but the targeting peptides are required for which the body could encode. The exosomal membrane was elucidated to contain multiple transmembrane proteins; among them, integrins, tetraspanins, lactadherin, and LAMP-2B are available for targeting modification purposes (Leidal et al., 2020; Ikeda et al., 2021). LAMP-2B is a member of lysosome-associated membrane proteins, which contain a large N-terminal extramembrane domain. Targeting peptides can be fused with the extracellular domain of LAMP-2B at the N-terminus through a gene editing technique (Thery et al., 2002). Rabies virus glycoprotein (RVG) peptides show selective binding to acetylcholine receptors and have been used to modify EVs to target the central nervous system (Liang et al., 2021). PDGFRα is highly expressed in the brain and spinal cord, and researchers overexpressed the PDGF ligand on EVs which fused on EV membrane protein MFG-E8, to improve the targeting efficiency of the central nervous system (El-Andaloussi et al., 2012). In addition, CD63 is a tetraspanin enriched on the exosome surface, CP05 binds specifically to CD63 (Xiao et al., 2022), and CP05 was applied to get EVs efficiently delivered to endothelial cells (ECs), thereby improving its therapeutic efficacy (Gao et al., 2018). As for cartilage targeting modification, Xu et al. developed a delivery system using the E7 peptide as a synovial fluid-derived mesenchymal stem cell-targeting peptide, which fused with LAMP-2B on the EV surface (Dong et al., 2021). In addition, CD47 is a transmembrane protein that enables cancer cells to evade clearance by macrophages, and EVs with CD47 overexpression were proved to keep EVs from phagocytosis by blocking macrophages in circulation, which promote targeting delivery to the infarcted myocardium in a roundabout way (Xu et al., 2021). But genetic methods can only link translatable peptides or segments onto specific transmembrane proteins, and the process was regulated internally by recipients that the proteins transport onto the membrane.

### Chemical modification

The surface of EVs can be modified by chemical modification in multiple methods, but the reactions are supposed to be mild and make the membrane bearable. For instance, the amine groups on EV surfaces can be easily modified with alkyne groups, which is also a commonly used method in EV modification. The alkyne-tagged exosomes can be coupled to azide-containing reagents through copper-catalyzed azide–alkyne cycloaddition (CuAAC) “click” reactions (Smyth et al., 2014; Wei et al., 2021) (Figure 1B). Wang et al. used alendronate (Ale)-N3 to modify MSC-EVs by copper-free “click chemistry” to generate an Ale-EV system, to target EVs to hydroxyapatite-enriched bone tissue, for specific osteogenesis (Poulsom, 1987). However, the reaction relies on the conversion of amine groups to alkynes, which is not specific, and the chemical modification that lacks site-specificity could block the common internalization process and prevent EVs from recipient phagocytosis.

Instead of non-specific binding, novel binding strategies are supposed to be raised. Amphipathic molecules such as polyethylene glycol (PEG) can be artificially inserted into the lipid bilayer of exosomes, and PEG-grafted 1,2-dioleoyl-sn-glycero-3-phosphoethanolamine (DSPE-PEG) is verified to accumulate in the exosome membrane. Li et al. used platelet membranes as a natural infarct-homing agent to target onto the myocardial infarction (MI) area, and DSPE-PEG acts as a catcher for dissociative palates in this system (Wang et al., 2020), which creates new possibilities for therapy in MI therapy.

### Non-covalent modification

In addition to covalent modification, non-covalent modification methods such as electrostatic interactions, ligand–receptor interactions, hydrophobic interactions, and aptamer-based surface modification are also being explored for targeted exosome production (Leidal et al., 2020) (Figure 1B). Exosomal membranes commonly bear negative potential, and scholars augmented cationed materials onto the exosomal surface *via* electrostatic interaction to improve the liver targeting capacity of the engineering vesicle (Tamura et al., 2017; Li et al., 2020). Wang et al. first attached biotin over human umbilical vein endothelial cell (HUVEC)-derived exosomes through avidin–biotin ligand–receptor interaction (Nakase and Futaki, 2015). Furthermore, hydrophobic interaction was utilized to fuse artificially manufactured liposomes and exosomes *via* the freeze-thaw method, which promoted the EV targeting capacity (Lee et al., 2016; Wang et al., 2017b). The linkage built through intermolecular forces seems to be not as reliable enough as the covalent ones, but the non-covalent connection methods have been used and come out to have preferable therapeutic effects.

## Biomaterials designed for MSC-EVs loading

Biomaterials are defined as materials which possess natural or artificial functions which are designed to contact and interact with living systems, and they are aimed at disease diagnosing, treating, replacing, repairing or inducing regeneration of cells, tissues, and organs. Biomaterials could be classified into multiple methods; according to their functionality, biomaterials generally include implants applied to deliver or undertake stress, artificial pumps which control the flow in the circulatory system such as prosthetic valves, artificial sensors with electricity, light, and sound conducting functions, and filling materials with the capacity to promote local tissue regeneration (Sato et al., 2016; Rupp et al., 2018; Oveissi et al., 2020). Due to their structural plasticity and machinability, biomaterials are more likely to act as scaffolds in tissue engineering. Intrinsic cells are recruited to the area’s biomaterials implanted for local regeneration, which is achieved by binding or loading growth factors on the scaffold, or the scaffold itself is suitable for cell cultivation and adhesion no matter *in vitro* or *in vivo*; both of the methods provide conditions and environments suitable for tissue regeneration, such as adequate blood supply, an anti-inflammation immune microenvironment, and a favorable habitat for stem cell expansion (Tavella et al., 2018; Li et al., 2021b).

In addition to facilitating regeneration, MSC-EVs have also been reported to protect against various diseases, yet low stability and retention in tissues restricted their exertion of reparative effects. MSC-EVs’ application in tissue regeneration acquires local concentration and stability maintenance. To maintain efficient local concentration and integrity of vesicles, researchers use timing quantitively injection, which could raise problems of EV accumulation and tissue injury (Bai et al., 2021). Nowadays, plentiful biomaterials are physically similar to the extracellular matrix (ECM), for instance, some of them are established by polymer fibers, which can form ordered bundles and microscopic pores, and provide space for cells to migrate in. Meanwhile, polymers can be conditionally depolymerized which creates possibilities for MSCs or vesicle-controlled releasing (Figure 1C).

### Metal scaffolds for EV loading

To achieve tissue regeneration *in situ*, the construction of biomaterials strives to match the requirement of structure and function. For example, for bone or joint defect repairing, scaffolds are required for mechanical stiffness, permissible deflection, and tissue compatibility. Metallic biomaterials are the prior choice which fit the stiffness requirement well. Titanium (Ti) implants and their alloy have been widely used in arthroplasty, craniofacial surgery, and orthodontic implant for decades (Zhang et al., 2022). Ti and its alloy bear both mechanical properties and biocompatibility, and they are essentially bio-inert and can hardly be corroded in an embedded microenvironment. Drawbacks such as insufficient osseointegration between metal and bone, aseptic loosening, shifting, or even detachment exist, probably because cells can barely attach or proliferate on the untreated metal surface, unless pre-roughening, coating, and finishing are carried out. In addition to that, inner bioactive components of MSC-EVs act as a regulatory role in osteointegration. MSC-EVs were previously demonstrated to be functioning in cell-based regeneration therapy, and they are validated as crucial in cell proliferation, migration, and immunoregulation, which perform a combining but overall positive role. Researchers recently use metallic scaffold coupling with EVs, which eventually raise the regenerative effect (Souza et al., 2019). Researchers even come up with strategies that coating EVs onto the metal surface to enhance the integrity of the interfaces between metal and bone. They pre-treated Ti discs by polishing, alkaline treatment, and plasma activation before co-cultivating with EVs, and EVs were covalently bound to the Ti surface through the layer of nanostructured sodium hydrogen titanate. The EV coating method turns out to significantly promote cell proliferation and Ca/P deposition on the Ti surface (Wu et al., 2020).

Titanium nanotubes (TNs), as a novel reservoir for EV loading, possess control-released capacity (Pansani et al., 2021). TNs generated on Ti implants are considered a novel and important modification technique. Zhao et al. incorporated EVs with titanium nanotubes generated on Ti implant surfaces, which consequently promoted MSC migration, macrophage adhesion and proliferation, and induced macrophage M2 phenotype polarization (Wang et al., 2017c) (Figure 1C).

Ti scaffolds were widely applied in sclerous tissue repair on account of their excellent stiffness and affinity (Zhao et al., 2021), but the elastic moduli of metal cannot match that of sclerous tissue of the human body (Wang et al., 2018), which caused the micro-leakage and accumulation of stress in the interface, and although they can perfectly ensure that the defect is filled and propped up, the long-term therapeutic effect is not satisfying (Arifvianto et al., 2017). The application of MSC-EVs provides a novel idea for the application and development of metal scaffolds.

### Inorganic scaffolds for EV loading

Compared with metal, inorganic materials can match the elastic moduli of hard tissue and meet mechanical strength requirements as well. Research studies have confirmed the significant potential of the calcium phosphate family [i.e., hydroxyapatite, *β*-tricalcium phosphate (TCP), whitlockite, etc.] in bone tissue regeneration, and porous calcium phosphate scaffolds have been widely used in bone defect repair (Bosshardt et al., 2017). Chai et al. (2012) and Qi et al. (2016) have co-incubated MSC-EVs with commercial *β*-TCP scaffolds to achieve EV attachment, which turns out to be effective for bone regeneration in the rat cranial defect model (Figure 1C). In addition to that, bio-glass and bio-ceramics are supposed to be novel carriers for EVs. Zhang et al. (2016) established a hierarchical mesoporous bioactive glass loaded with MSC-EVs, and the system was designed to retain and release MSC-EVs, which targeted rat bone marrow stromal cells (rBMSCs). The scaffold showed a burst EV release in the first week followed by a steady slow release, and 28 days later, the scaffold still retained some EVs by its microporous structure, which differed from current strategies for vesicle entrapment and retaining, and the scaffold ultimately showed brilliant osteogenic effects. Inorganic scaffolds cannot generally be absorbed and remodeled by the body and can barely achieve vascularized regeneration in local implanting positions. Appropriate porosity can indeed promote local vascularization for tissue regeneration (Karageorgiou and Kaplan, 2005; Liu et al., 2021), but according to the clinical requirement and the diverse shapes of tissue defects, injectable and absorbable scaffolds are more commonly needed.

### Scaffolds fabricated by macromolecules for EV loading

Compared with inorganic biomaterials, biomedical polymer materials attract more attention due to their superior plasticity, and the cross-link between different molecules opens up many possibilities for the construction of materials. As the design and craft of biomaterials advance, high precision and efficiency are necessary requirements for clinical transformation. EV-controlled release can be achieved *via* carriers which are designed and established on purpose, and the composite scaffolds can meet the requirement of conditional tissue regeneration, which is supposed to be perseverant and tissue-specific (Barba et al., 2018; Wang et al., 2019). There are mainly the following forms of scaffold established for EV retaining and control-releasing.

As for tissue regeneration scaffolds, ECM fabricated with collagens, proteoglycans/glycosaminoglycans, elastin, fibronectin, laminins, and several other glycoproteins, is the most preferable choice for cell adhesion, proliferation, and stretching, which provides a suitable microenvironment for tissue regeneration (Shi et al., 2021). Hydrogels are cross-linked 3-dimensional polymeric networks, which play an important role in tissue regeneration application (Figure 1C). As excellent alternatives originate from ECM, hydrogels mimic ECM in structures and functions. An ideal hydrogel scaffold is supposed to have appropriate mechanical properties, good water retention, anti-infection capacity, injectable capacity, and good cell biocompatibility, and they can be simply be divided into natural and synthetic in material origin (Theocharis et al., 2016). Hyaluronic acid synthesis pioneer bio-hydrogels which basically originate from decellularized ECM, are a natural hydrogel and mostly derived from polypeptides (e.g., fibrin, collagen, and gelatin) and polysaccharides (e.g., hyaluronic acid, alginate, cellulose, and chitosan). Synthetic hydrogels [e.g., poly (ethylene glycol), poly (acrylate) derivatives, poly (methacrylate) derivatives, poly (acrylamide), poly (lactic-co-glycolic acid), poly (vinyl alcohol), and poly (urethane).] possess high tunability during synthesis; since the ratio of polymer molecules, crosslinking ratios, and synthesis conditions can affect the final physical and chemical properties of the gel, different material choices govern the gel biocompatibility, porosity, degradability, and speed of EV release (Gradinaru et al., 2018). EVs can be loaded onto hydrogel scaffolds by multiple methods; the first is by adding crosslinking agents to the polymer solution containing EVs to form the available hydrogel. Qin et al. used polyethylene glycol diacrylate (PEGDA) as a gelation agent to construct a hyaluronic acid-based system containing BMSC-derived EVs, and the nanoscale EVs were stuck in the chamber fabricated (Hu et al., 2020). The second is that the freeze-dried hydrogel “breath-in” the EVs contained aqueous to form an EV-loaded scaffold called cryogel (Qin et al., 2016; Eggermont et al., 2020). Cryogel is validated to be a better scaffold qualified for bone and cartilage regeneration, owing to its larger porosity and pore volume. Nikhil et al. demonstrated that the cryogel had a promising prospect as a tissue regenerative scaffold (Razavi et al., 2019). The last is incorporating EVs with both the polymers in the solution and crosslinkers simultaneously, which is called *in situ* gelation (Tavella et al., 2018). Zhang et al. demonstrated that the integrins increased the stability of EVs within hydrogels, and the researchers incubated EVs with the hydrogel at 37°C for 8 h, which established solid binding (Barba et al., 2018).

Hydrogel scaffolds are widely used in skin wounds (Nikhil and Kumar, 2022), periodontitis, and several defects in soft tissue due to their excellent fluidity, sustained release capacity, and suitable porosity. For instance, periodontitis is a peculiar inflammatory disease, which is often accompanied by bacterial infiltration defects, either in gingiva or bone, but dentists prefer bone regeneration rather than inflammatory soft tissues. The loaded MSC-EVs can reverse the unfavorable inflammatory microenvironments and promote bone regeneration. For instance, Shen et al. (2020) fabricated a chitosan hydrogel incorporated with dental pulp MSC-derived EVs, to create a favorable immune-environment by converting the phenotype of periodontal macrophages. Hydrogels possess favorable characteristics in tissue regeneration, but they are almost easily depolymerized in the unwanted sites, and commercial hydrogels can hardly conditionally release in specific defect areas while retaining vesicles in others. Some macromolecules owing to their conditional cross-linking and depolymerization properties have been applied in specific scenarios. For example, photocrosslinkable gelatin methacryloyl (GelMA) hydrogel originated from the modification of amine-containing side groups of gelatin (Gel) with methacrylamide and methacrylate groups; it owns the integrin binding motif, which makes it a suitable scaffold for cell adhesion, and GelMA has matrix metalloproteinase (MMP) degradation sites. MMP is highly expressed in inflammatory areas, and the role of MMP is to degrade inflammatory ECM. The MMP-specific degrading sites make GelMA a promising scaffold aimed at inflammatory tissue defects (Wells et al., 2015), and the hydrophilicity of GelMA makes it biocompatible for recipients (Zhang et al., 2016). However, the lack of mechanical strength of hydrogels has been criticized in clinical applications. Gradinaru et al. (2018) came up with a fascinating strategy of loading miR-23a-3p-abundant small extracellular vesicles in the GelMA hydrogel for cartilage regeneration. They introduced laponite nanoclay into the system in a pioneering way. It is believed that the introduction of such multi-functional scaffolds will bring new direction and progress for clinical transformation and application in tissue regeneration.

## Discussion

EVs, as natural cell by-products, play a very important role in the progression and prognosis of disease processes. EVs secreted by MSCs mirror the function of MSCs and play a pivotal role in tissue regeneration. EVs conjugated with biomaterials improve EV targeting capacities and stabilize EV residents in the target tissue, which promotes EV delivery efficiency. The EV-biomaterials’ combined strategy turns the clinical transformation of MSC-EVs into a possibility, and provides not only theoretical assistance for cell-free therapies in tissue regeneration but also direction for clinical practice.

As the secretion of MSCs, EVs were endowed with promising prospects in tissue engineering. EVs bring about opportunities and challenges simultaneously; the opportunity is that the nano-sized vesicles reach the defect where general drugs are inaccessible, such as the kidney, heart, and brain (Ribeiro et al., 2020; Dar et al., 2021). The challenge is that EVs can hardly maintain their effective concentration *in vivo* due to phagocytosis. Moreover, the easy-to-degrade character makes EVs difficult to store and maintain (Tang et al., 2020). In addition, EVs commonly possess targeting capacity (Zhang et al., 2020), but some tissues and organs are difficult to realize regeneration *via* EVs’ local applications. Therefore, engineering biomaterial construction of EVs is necessary. On the one hand, engineering EVs can increase the retention rate by blocking phagocytosis; on the other hand, the engineering modification can improve the targeting ability and enhance regenerative efficiency caused by the accumulation in the liver and kidney, correspondingly improving the uptake of the target tissue (Vader et al., 2016).

We emphasized the engineering modification of EVs and their carrier construction, and researchers have come up with strategies for changing EV surface ligands, EV components, and EV carriers for further enhancing the EV control-releasing and targeting capacity, thus improving the specificity in regeneration. We briefly summarized the research progress and advancing direction of MSC-EVs engineering in recent years, which mainly covers EV-coupled scaffolds and EV-surface modification. Although the amount of issues has been boosted since emerging, there still are plenty of problems demanding prompt solution in engineering EV construction; one is that the regenerative and targeting effect is relatively verified, and there are rarely pieces of evidence for space-temporal consistence in EV-associated biomaterials.

As mentioned previously, surface engineering is a key technical method for EV modification, but in the process of bonding the targeted part to the vesicle surface, some functional membrane proteins (such as histocompatibility antigens and heat shock proteins) may be blocked and the integrity of the vesicle membrane may be damaged as well, which results in the loss of functional components. In addition, chemical operations may cause changes in the surface charge of the EVs, lead to the agglomeration of the vesicles, the adsorption of proteins, and undesirable deposition and clearance, all of which may conceal the targeted molecules *in vivo*. The crosslinking conditions of the scaffolds that load the vesicles may also damage the overall structure of the vesicles. Researchers pay more attention to the source and targeting ability of engineered vesicles, but little attention is paid to the ultimate destination and final metabolites of these engineered vesicles, whether they can be decomposed and metabolized by the body, and whether the engineered EVs will burden the metabolic system, are rarely investigated. As we explore in our research, genetic surface modification of EVs is preferred for its higher efficacy and biosecurity. Disintegration of EVs and their content loss are ongoing during *in vitro* storage and reaction, and the prolonged engineering process is detrimental to the therapeutic efficacy of EVs.

Above all, MSC-EVs are promising methods for tissue regeneration, and the associated biomaterials and engineering methods act as support for the *in vivo* application of EVs. The purpose of this review is to look inward and move forward for EVs’ development, which further shed new light and provide indications for the rational design of EV-associated biomaterials’ efficiency and applied engineering EVs.
